# MORTALITY PREDICTOR IN ELDERLY PATIENTS AFTER PROXIMAL THIRD FEMUR FRACTURE: ANALYSIS OF 395 CASES

**DOI:** 10.1590/1413-785220253305e289995

**Published:** 2025-09-22

**Authors:** Jean Klay Santos Machado, Eduardo Cezar Silva dos Santos, Deivid Ramos dos Santos

**Affiliations:** 1 Hospital Porto Dias, Departamento de Ortopedia e Traumatologia, Belem, PA, Brazil.

**Keywords:** Aged, Mortality, Risk Factors, Femur, Fractures, Bone, Idoso, Mortalidade, Fatores de Risco, Fêmur, Fraturas Ósseas

## Abstract

**Objective:**

To assess the risk factors associated with mortality in elderly patients with proximal femoral fractures up to one year after the fracture.

**Method:**

This was a prospective cohort study of patients aged 60 or older diagnosed with a proximal femoral fracture at a referral hospital in Belém, PA.

**Results:**

395 patients were assessed. The risk factors age≥83 years (p<0.0010), fracture-surgery time ≥ 3 days (p=0.0034), hemoglobin < 9g/dL (p<0.027), leukocytosis (p<0.00001) and pulmonary infection (p<0.0001) were statistically significant in mortality up to 12 months after the fracture.

**Conclusion:**

It was observed that in the first year after fracture, gender, type of fracture and comorbidities were not determining factors in mortality. In this series, the mortality rate was 20.75% one year after the fracture, with pulmonary sepsis being the most frequent cause of death. ***Level of Evidence II; Cohort Study.***

## RESUMO

**Objetivo:**

Avaliar os fatores de risco associados a mortalidade de pacientes idosos com fratura da extremidade proximal do fêmur em até 1 ano após a fratura.

**Método:**

estudo de coorte prospectivo e analítico, em pacientes com idade superior ou igual a 60 anos diagnosticados com fratura da extremidade proximal do fêmur atendidos em um hospital de referência em Belém-PA.

**Resultados:**

Foram avaliados 395 pacientes. Os fatores de risco idade≥83 anos (p<0,0010), tempo fratura-cirurgia ≥ 3 dias (p=0,0034), hemoglobina < 9g/dL (p<0,027), leucocitose (p<0,00001) e infecção pulmonar (p<0,0001) foram estatisticamente significantes na mortalidade em até 12 meses após a fratura.

**Conclusão:**

Foi observado que no primeiro ano pós fratura o gênero, tipo de fratura e a comorbidades não foram fatores determinantes na mortalidade. Nesta casuística, a taxa de mortalidade foi de 20,75% após um ano da fratura, sendo a sepse de foco pulmonar a causa mortis mais frequente. ***Nível de Evidência II; Estudo de coorte.***

## INTRODUCTION

Proximal femur fractures are common in older people, with an estimated 6 million cases in 2050.^1^ They can account for up to 84% of bone injuries in people over 60 worldwide.^2^ When they occur, these fractures are associated with high morbidity and mortality rates, social impact, and high hospital costs involved in patient care.^1^ It should be noted that the increase in the incidence of fractures in this segment is primarily influenced by demographic transition, with an increase in the number of elderly individuals reaching extreme ages due to advances in medicine and improvements in the population's quality of life.^3,4^ In Brazil, for example, it is estimated that by 2060, there will be more elderly people than young people.^5^ These fractures are usually treated surgically with total or partial prostheses or osteosynthesis, and even with technical and therapeutic advances, they still have a high morbidity and mortality rate.^6–8^ Despite this, and even though Brazil has regional differences and unique characteristics in each state, especially in the North, there is no predictive model of mortality for patients with femur fractures based on this population profile.

It is well documented that the presence of associated comorbidities, such as pulmonary infection, pressure ulcers, and bed rest associated with embolisms, has devastating effects on patients with this type of fracture,^9,10^ making it necessary to conduct studies on the morbidity and mortality of elderly patients with femoral fractures in the North of Brazil.

Thus, the objective of this study is to analyze the factors associated with mortality in patients over 60 years of age who underwent surgical treatment for proximal femur fractures, to identify predictors of mortality.

## METHOD

This is a prospective cohort study based on medical records from Hospital Porto Dias, Belém do Pará, Brazil, a regional reference center for orthopedics and traumatology. The study was approved by the Research Ethics Committee of the State University of Pará, under number 2,724,974, protocol CAAE 91604318.8.0000.5167. All elderly patients who underwent surgical treatment for fractures in the proximal third of the femur (neck, intertrochanteric, and subtrochanteric fractures), well documented by imaging tests, were included in the study, selected through convenience sampling. All patients aged 60 years or older were considered elderly. Patients with duplicate medical records, associated fractures in other body segments, and those treated non-surgically (n = 120) were excluded from the study. The study included 395 cases that were followed up for 1 year.

The variables defined were age, gender, time interval between fracture and surgery, type of surgery, red blood cell indices on admission (hemoglobin and leukocytes), postoperative complications, associated comorbidities, occurrence of death up to 1 year after fracture, and cause of death (when applicable), using a code from the International Statistical Classification of Diseases and Related Health Problems (ICD-10).

To minimize the risk of bias, the date and cause of death were confirmed by telephone contact with the families of patients who had such an outcome. A researcher recorded all data collected and independently triple-checked.

Statistical inference was implemented through hypothesis testing. Initially, the Odds Ratio test (Student's t-test or Mann-Whitney test) was applied, and factors with a significance level of less than 0.05 were submitted to bivariate logistic regression. The alpha significance level was previously set at 0.5 for rejection of the null hypothesis.

Microsoft Office software was used to prepare data, tables, and texts, and Bioestat 5.4 was used for qualitative statistical analysis.

## RESULTS

Of the 395 patients included in this study, 20.75% (n = 82) died within 12 months after the fracture. Of these, 18.3% of patients in the death group and 18.2% in the survival group were male, with a p-value of 0.88. Thus, gender is not a statistically significant factor for mortality in patients with proximal femur fractures. In absolute numbers, there are way more women than men.

When analyzing the age group, it was observed that 58.5% (n = 48) of patients in the death group were aged 83 years or older, whereas only 37.7% (n = 118) of patients in the survival group were in this age group. The chance of a patient over 83 years of age dying was 2.4 times higher than patients under that age, and the mortality rate in the over-83 age group was 28.9% within 12 months of the fracture. Therefore, age is a statistically significant risk factor for mortality (p = 0.0010).

Considering the interval between fracture and surgery, a statistically significant difference was observed (p = 0.0034), as the mortality rate of patients treated after 3 days was 28.6%, compared to patients who were treated within 48 hours, in whom this percentage was 15.8%.

As observed in Table 1, although transtrochanteric fractures were the most common in absolute numbers, the type of fracture was not a determining factor for patient mortality (p = 0.4033). Similarly, the type of surgical treatment is not a determining factor for mortality in these patients (p = 0.731), with no statistically significant difference observed between the types of treatment.

**Table 1:** Assessment of the risk of death among 395 patients treated at a referral hospital in Belém, Pará, 2024.

	Death		Survival			Odds Ratio		
Risk Factor	(n=82)	%	(n=313)	%	p-value	OR	95% CI	Mort.%
**Sex**					**0.8858**	**1.1**	**0.5 to 1.8**	
Male	15	18.3	57	18.2				20.8
Women	67	81.7	256	81.8				20.7
**Age group**					0.0010*	**2.4**	**1.4 to 3.8**	
83 years old or older*	48	58.5	118	37.7				28.9
60 to 82 years old	34	41.5	195	62.3				14.8
**Fracture-surgery time**					0.0034*	**2.1**	**1.3 to 3.5**	
3 days or more*	44	53.7	110	35.1				28.6
Up to 2 days	38	46.3	203	64.9				15.8
**Type Surgery**					**0.7361**	**1.1**	**0.7 to 1.8**	
DCS	4	4.9	24	7.7				14.3
DHS	5	6.1	20	6.4				20.0
HASTE	50	61	182	58.1				21.6
Arthroplasty*	16	19.5	87	27.8				15.5
**Hemoglobin**					0.0027*	**2.5**	**1.4 4.3**	
HB < 9.0*	24	29.3	45	14.4				34.8
HB >= 9.0	58	70.7	268	85.6				17.8
**White blood cells**					<0.00001*	**5.1**	**3.0 to 8.6**	
Leukocytosis	49	59.8	70	22.4				41.2
No leukocytosis	33	40.2	243	77.6				12.0
**Complications**					<0.0001*	**5.6**	**3.0 to 10.1**	
Lung infection*	29	35.4	28	8.9				50.9
Absence	53	64.6	285	91.1				15.7
**Comorbidity AS**					**0.3019**	**1.4**	**0.8 to 2.5**	
HAS present	63	76.8	220	70.3				22.3
Absence	19	23.2	93	29.7				17.0
**Fracture type**					**0.4033**	**1.2**	**0.7 to 2.2**	
Colo	21	25.6	93	29.7				18.4
Subtrochanteric	8	9.8	36	11.5				18.2
Transthoracic*	53	64.6	184	58.8				22.4

Source: Research protocol.

Regarding red blood cell indices, it was observed that patients with hemoglobin levels below 9 g/dl had a 2.5 times higher risk of mortality than patients with hemoglobin levels above 9 (p = 0.0027). With regard to leukocyte count, patients with leukocytosis are 5.1 times more likely to die than patients with normal leukocyte indices. The complication of pulmonary infection is a significant risk factor, given the 5.6-fold increase in mortality compared to patients who did not develop it.

Systemic hypertension, despite being the most frequent in the case series, was not statistically significant when analyzing the outcome (p = 0.319).

Table 2 shows that Evans unstable fractures were more prevalent in both the death group (54.9%) and the survival group (44.7%). However, it was not statistically significant (p = 0.2508) as a direct risk factor for patient mortality within twelve months.

**Table 2:** Analysis of the classification of neck fractures (Garden), transtrochanteric fractures (Evans), and subtrochanteric fractures (Sensheimer) in 395 patients treated at a referral hospital in Belém, PA, 2024.

	Death		Survival		
	N (82)	%	N (313)	%	p-value
**Garden**					**0.8961**
With deviation	17	20.7	74	23.6	
Without deviation	3	3.7	15	4.8	
NSA	62	79.2	224	71.6	
**Evans**					**0.2508**
Stable	8	9.8	43	13.7	
Unstable	45	54.9	140	44.7	
NA	29	35.4	130	41.5	
**Sensheimer**					**0.1938**
IIA	0	0.0	7	2.2	
IIB	4	4.9	10	3.2	
IIC	0	0.0	3	1.0	
IIIA	0	0.0	4	1.3	
IIIB	0	0.0	6	1.9	
IV	1	1.2	1	0.3	
V	3	3.7	4	1.3	
NSA	74	90.2	277	88.5	

Source: Research protocol.

**Figure 1:**
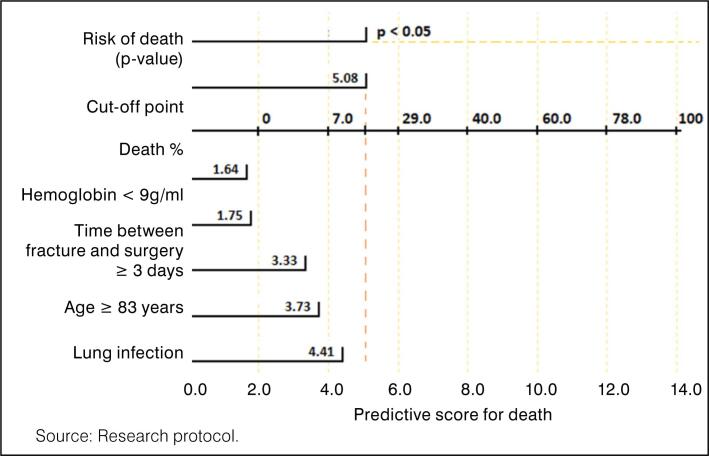
Nomogram predicting death among 395 patients treated at a referral hospital in Belém, Pará, 2024.

The multivariate model showed the predictive variables that had proven significance by logistic regression: age ≥ 83 years (OR = 3.33), fracture-surgery time ≤ 2 days (protective factor OR = 1.75), hemoglobin < 9 (OR = 1.64), leukocytes ≥ 10,000/mm^3^ (OR = 1.48), and pulmonary infection (OR = 1.32), as shown in Table 3 and Figure 1.

**Table 3:** Multivariate logistic regression model applied to 395 patients treated at a referral hospital in Belém, PA, 2024.

Variable	Coefficient	p-value	Odds ratio	95% CI
Intercept	-3.15			
Age ≥ 83	1.20	<0.0001*	3.33	1.89 to 5.89
Time between fracture and surgery ≥ 3	0.56	0.0485*	1.75	1.10 to 3.07
Hemoglobin < 9	0.49	0.1404	1.64	0.85 to 3.20
Leukocytosis	1.48	<0.0001*	4.41	2.50 to 7.78
Lung infection	1.32	0.0001*	3.73	1.91 to 7.30

Source: Research protocol.

Based on the logistic regression data, it was possible to develop a nomogram predicting death in elderly patients with femur fractures. In the nomogram, each risk factor is assigned a score: hemoglobin (1.64 points), age (3.33 points), pulmonary infection (3.73 points), leukocytes (4.41 points), and time elapsed between fracture and surgery (1.75 points). The sum of the points for the six factors yields the overall score on the Nomogram, with a cutoff point of 5.08. If this value is reached, the probability of death is statistically significant (p-value < 0.05). This score shows Sensitivity = 0.728 (72.8%), Specificity = 0.720 (72.0%), and Accuracy = 0.726 (72.6%), as shown in Figure 02.

**Figure 2:**
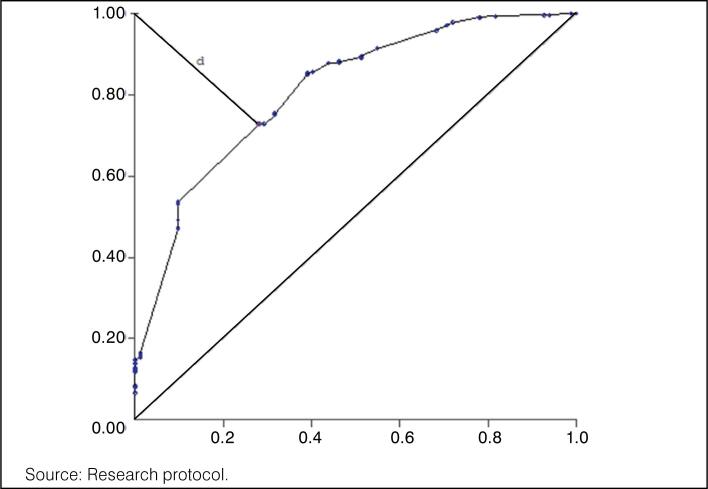
ROC curve of the death prediction nomogram for 395 patients treated at a referral hospital in Belém, PA, 2024.

## DISCUSSION

Fractures of the proximal third of the femur are associated with a high mortality rate.^2,3^ In an attempt to mitigate this situation, several institutional protocols have been developed, primarily based on risk stratification tools, to identify key factors associated with poor prognosis and to prevent them.^5^ Thus, this study creates a simplified mortality predictor based on initial data acquired upon patient admission.

Considering the significant physiological impact caused by proximal femur fractures, there is a high incidence of death in the first year after the fracture. Studies show mortality rates ranging from 12% to 37% in one year and up to 50% in two years, which is quite similar to the findings of this study, which included 20.75 cases (n=82).^11–13^ This high global mortality rate is justified by the decline in physical function in the elderly population, underlying diseases, and complications associated with the tissue trauma resulting from the fracture itself. This generates pathophysiological responses caused by inflammatory reactions that exacerbate inflammatory responses and, in some cases, can trigger systemic inflammatory response syndrome (SIRS) and multiple organ failure, especially when the patient already has underlying cardiopulmonary disease.^11^

In this study, no statistically significant difference was found between genders. However, in absolute numbers, females were proportionally higher, given the greater number of women who reach older ages compared to males, a finding consistent with other studies.^2^ It is worth noting the possibility of osteoporosis developing in postmenopausal women and the longer life expectancy of women, which is a risk factor for a higher incidence of fractures in this population.^14,15^ There is a strong association between mortality in patients over 83 years of age.^16,17^ As age increases, the ability to withstand trauma, anesthesia, and surgical treatment itself decreases, favoring the development of complications that contribute to death.

In our study, we found that the group of patients treated surgically within 48 hours of admission had a 1-year mortality rate of 15.8%, compared to patients who underwent surgery after this time, with a mortality rate of 28.6% (p < 0.0001). It can be said that delay in surgical treatment contributes to an increased risk of thrombosis, bronchoaspiration pneumonia, systemic changes in decompensated diseases, among other factors. It has been identified that for every 12 hours of delay in definitive treatment, there is a 7% increase in the risk of mortality within 12 months.

Some studies argue that the best period for surgical treatment is within the first 36 hours after admission, with a direct impact on reducing mortality in the short and medium term^10^. It has been identified that for each year following surgical treatment, there is a 9.4% increase in the risk of death, corroborated by the greater vulnerability of this population.

Unfortunately, the clinical instability and advanced age of some patients require intensive care beds so that the patient can be stabilized and discharged for the definitive procedure. On the other hand, this often delays surgical treatment and triggers a higher risk of bronchoaspiration pneumonia and hospital infection.^17–19^

The fracture pattern (p = 0.4033) and type of surgical treatment (p = 0.731) were not determining factors for mortality in the patients evaluated in this study. Further studies with a larger number of participants are required to increase statistical reliability. Despite this, some studies agree with our results.^20,21^

In this study, patients with severe anemia were associated with a 2.5-fold higher risk of mortality compared to patients with hemoglobin levels above 9 (p = 0.0027).^22,23^ Alteration of this red blood cell indices implies a poor prognosis given its association with various comorbidities, especially in patients at extreme ages with a certain degree of documented chronic disease anemia.^22^

When suffering a fracture of the proximal end of the femur, there is a small drop in hemoglobin levels between admission and surgery (average of 0.3 g/dL). In contrast, the average drop in hemoglobin after surgery was considerable (average of 2.8 g/dL), roughly equivalent to a loss of 2 to 3 units of blood. This decline is justified due to bleeding resulting from surgical treatment and hemodilution caused by intravenous fluids administered since the patient's admission to the hospital.

The degree of anemia is a strong and independent prognostic factor because it is a marker of underlying disease burden.^14^

In this study, leukocytosis was associated with a fivefold increase in the risk of death compared to patients without this laboratory abnormality at admission. Some studies have indicated that neutrophils are most closely associated with mortality.^1–3,17,18^ In other words, elevated neutrophil counts reflect long-term prognosis.

It should be noted that other studies also report lymphopenia related to death resulting from late complications such as surgical site infection, pneumonia, and sepsis. Leukopenia is related to delayed and poor wound healing after a fracture, enabling the growth and proliferation of microorganisms that contaminate the surgical wound, constituting an important risk factor for postoperative sepsis and mortality.

In the nomogram, each risk factor is assigned a score: hemoglobin (1.64 points), age (3.33 points), pulmonary infection (3.73 points), leukocytes (4.41 points), and time elapsed between fracture and surgery (1.75 points). The sum of the points for the six factors yields the overall score on the Nomogram, with a cutoff point of 5.08. If this value is reached, the probability of death is statistically significant (p-value < 0.05). This score shows Sensitivity = 0.728 (72.8%), Specificity = 0.720 (72.0%), and Accuracy = 0.726 (72.6%), as shown in Figure 02.

It is essential to note that risk identification models are not a substitute for initial medical assessment, which remains a crucial factor in clinical and surgical decision-making. When advising patients with femur fractures and their families about the prognosis, or when deciding on surgical or palliative strategies, medical assessment may be more valuable than these models. Future studies should include clinical evaluation when investigating mortality prediction in elderly patients with proximal femur fractures.^10–17^

Nevertheless, it is important to note that early assessment of individual risk factors is crucial for the proper management of this type of injury and, consequently, for reducing mortality. As a limitation, we note that this study is based on a sample from a single reference center, which is a relatively small sample, and further studies are needed.

## CONCLUSION

In this study, a mortality predictor for patients with proximal femur fractures was developed using hospital admission data. In a cohort of 395 patients evaluated, neither gender nor fracture type was identified as a significant risk factor for patient mortality. The time interval between fracture and surgery, hemoglobin level < 9.0 g/dL, leukocytosis, and age were directly associated with mortality in patients within 12 months after fracture as risk factors.
